# Clinical and histopathological characterization of metastatic lobular breast cancer: lessons learned from post-mortem tissue donation programs

**DOI:** 10.1038/s41523-026-00912-5

**Published:** 2026-02-20

**Authors:** Gitte Zels, Karen Van Baelen, Alexander CC Chang, Anirudh Pabba, Maxim De Schepper, Marion Maetens, François Richard, Josephine Van Cauwenberge, Tatjana Geukens, Kristien Borremans, Amena Mahdami, Ha Linh Nguyen, Sophia Leduc, Patrick Neven, Hans Wildiers, Vincent Vandecaveye, Raphaëla Dresen, Wouter Van Den Bogaert, Rohit Bhargava, Tanner Bartholow, Neil Carleton, Ye Cao, Jie Bin Liu, Abdalla Wedn, Hunter Waltermire, Morgan Cody, Lori Miller, Margaret Q. Rosenzweig, Julia Foldi, Marija Balic, Christoper Deible, Christine Hodgdon, Stephanie Walker, Adrian V. Lee, Steffi Oesterreich, Giuseppe Floris, Christine Desmedt

**Affiliations:** 1 Laboratory for Translational Breast Cancer Research, Department of Oncology, KU Leuven, Leuven, Belgium; 2 Department of Pathology, University Hospitals Leuven, Leuven, Belgium; 3 Department of Gynaecology and Obstetrics, University Hospitals Leuven, Leuven, Belgium; 4 UPMC Hillman Cancer Center, Pittsburgh, PA USA; 5 School of Medicine, University of Pittsburgh, Pittsburgh, PA USA; 6 Department of General Medical Oncology, University Hospitals Leuven, Leuven, Belgium; 7 Department of Radiology, University Hospitals Leuven, Leuven, Belgium; 8 Department of Forensic Medicine, University Hospitals Leuven, Leuven, Belgium; 9 Department of Pathology, University of Pittsburgh School of Medicine, Magee-Womens Hospital, UPMC, Pittsburgh, PA USA; 10 School of Medicine, Tsinghua University, Bejing, China; 11 Magee-Womens Hospital, UPMC, Pittsburgh, PA USA; 12 National Surgical Adjuvant Breast and Bowel Project (NSABP), Pittsburgh, PA USA; 13 Department of Radiology, UPMC, Pittsburgh, PA USA; 14 Guiding Researchers and Advocates to Scientific Partnerships (GRASP Cancer), Baltimore, MD USA; 15 Metastatic Breast Cancer Alliance, New York City, Ny, USA; Living Beyond Breast Cancer (LBBC), Bala Cynwyd, PA USA; 16 Institute for Precision Medicine, UPMC, Pittsburgh, PA USA; 17 Department of Imaging & Pathology, Laboratory for translational Cell & Tissue Research, Leuven, Belgium

**Keywords:** Cancer, Oncology

## Abstract

While primary invasive lobular carcinoma (ILC) is well characterized, metastatic ILC remains understudied. Within the post-mortem tissue donation programs, UPTIDER (Belgium) and Hope for Others (USA), we first aimed to explore intra-patient heterogeneity of key prognostic and predictive markers (stromal tumor-infiltrating lymphocytes (sTIL), estrogen receptor (ER), progesterone receptor (PR), human epidermal growth factor receptor 2 (HER2) and KI67). Secondly, we compared detection of the metastases by pathology on autopsy samples versus pre-mortem imaging. In total, 306 metastases from 12 patients were collected at autopsy (median: 27 per patient). Both primary tumors (*n* = 15) and metastases (*n* = 232) had low sTIL levels, with a median of 2% (range: 0.67–6.67%) and 0.67% (range: 0–13.33%), respectively. Regression models showed lower ER- and PR-expression in metastases (respectively, *n* = 265 and *n* = 64) compared to primary tumors (both p < 0.01). KI67 was significantly higher in metastases (*n* = 262, *p* = 0.02). HER2-low metastases were found in all but one patient although in varying proportion of metastases (range: 7.5–100%). Central radiology and pathology review had a median concordance of 78% at organ level (range: 33.33–100%) and 71% at patient level (range: 55.88-85.29%). Our findings suggest that a single metastatic biopsy has great limitations to guide treatment and that more adequate methods are needed to detect and monitor ILC metastases.

## Introduction

Invasive lobular carcinoma (ILC) is the second most common histological subtype of breast cancer (BC) after invasive breast cancer of no special type (IBC-NST), accounting for approximately 15% of all BC diagnoses^1^. Compared to IBC-NST, ILC frequently metastasizes to atypical sites, such as the gastrointestinal tract, orbit, and reproductive organs, as well as the more typical metastatic BC sites like bone and liver^2–5^. ILC is less often associated with metastases to, amongst other, lung and brain, although leptomeningeal disease seems to be more prevalent in patients with ILC^5–7^.

While histopathological differences between primary and metastatic lesions are well described for hormone receptor-positive BC in general^8^, they are poorly documented for ILC. In the retrospective study from Trillo et al.^9^, matched primary and metastatic tumor samples from 91 patients with ILC were evaluated and showed a discordance in receptor status in 15%, 44% and 5% of the patients for estrogen receptor (ER), progesterone receptor (PR) and human epidermal growth factor receptor 2 (HER2), respectively. The comparison was however limited to two metastatic samples for most of the patients.

The visualization of metastatic ILC by conventional imaging is challenging because of the typical discohesive cell growth of ILC without mass formation, leaving the normal architecture of the organ unaltered^10–12^. For example, the metastatic spread to the peritoneum is hard to distinguish on standard computed tomography (CT)^13^. Although new positron emission tomography (PET) modalities and whole-body diffusion weighted magnetic resonance imaging (WB-DWI/MRI) have shown improved detection of ILC metastases, they are not globally integrated into clinical practice^12–16^.

Histopathological assessment remains the gold standard to confirm presence of ILC metastases, however this is not always clinically feasible or justified^17,18^. Lesions might not be accessible for biopsy because of their small size, their anatomical localization or their proximity to vital structures. Additionally, due to the invasive procedure, the number of biopsies is limited to a minimum^18^. This implicates that these few biopsies might not be representative of other metastases that are present at that time.

The aforementioned hurdles and challenges hamper research on metastatic ILC. Post-mortem tissue donation programs, using rapid autopsies, provide access to a much larger sample size of metastases within the same patient and provide opportunities to extensively evaluate intra-patient heterogeneity of routinely assessed histopathological features^18,19^. Such programs have the ability to enhance the research of metastatic ILC. This study, which is based on patients with metastatic ILC who underwent a rapid autopsy in the context of two post-mortem tissue donation programs, had two main objectives: 1) to explore intra-patient heterogeneity of the key biomarkers stromal tumor-infiltrating lymphocytes (sTIL), ER, PR, HER2, KI67, and, 2) to compare detection of the metastases by pathology on autopsy samples versus pre-mortem imaging.

## Results

### Overview of the clinicopathological characteristics

**Fig. 1:**
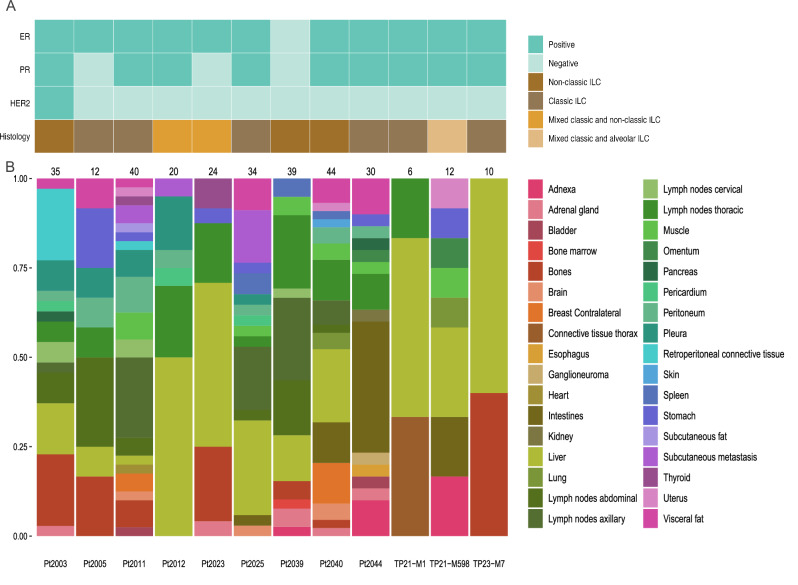
Clinical characteristics and metastatic spread of the patients. **A** Visualization of the main clinical characteristics (ER, PR and HER2) and histological ILC subtype of the primary tumor per patient. Dark green indicates receptor positivity (ER and PR > 1% and HER2 2+ or 3 + ISH positive) and light green indicates receptor negativity. **B** Metastatic spread in each patient with the number of metastases per patient indicated above the histogram and the different colors representing the various organs involved as indicated by the accompanying legend. ER: estrogen receptor; PR: progesterone receptor; HER2: human epidermal growth factor receptor 2; ILC: invasive lobular breast cancer.

Post-mortem tissue donations were performed on 12 patients who were diagnosed with primary ILC (9 patients in UPTIDER, 3 patients in HfO). The main characteristics of the primary tumor are shown in Fig. 1A and patient characteristics in Table 1. The primary tumor was composed of classic ILC in 50% of all patients, mixed classic and non-classic ILC in 3 patients (25%), pleomorphic ILC in 2 patients (17%) and 1 patient with histiocytoid/apocrine ILC (8%) (Fig. 1A, Supplementary Table 1). Most patients were diagnosed with ER-positive and/or PR-positive and HER2-negative disease (n =10 ). Patient 2039 (Pt2039) was diagnosed with a triple negative primary histiocytoid/apocrine ILC. The median age at primary invasive diagnosis was 49.5 years (range: 37-83 years). Half of the patients presented with de novo metastatic disease, while the other half had a median distant recurrence free survival of 154 months (range: 55-358 months). The median overall survival was 81 months (range: 15-427 months). Detailed patient characteristics can be found in Supplementary Table 1. The median post-mortem interval (time between death and start of post-mortem tissue donation procedure) was 3.3 hours (range: 1.8-5.9 hours). The post-mortem tissue donation procedures from UPTIDER and HfO yielded a total of 306 metastases with a median of 27 metastases per patient (range: 6-44 metastases). The fixation time varied from 1 day up to 33 days. Additionally, we were able to retrieve untreated primary tumor samples for 9 patients.

**Table 1:** Patient characteristics

		UPTIDER (n = 9)	HfO (n = 3)	TOTAL (n = 12)
Diagnosis	**Age at diagnosis**			
	< *50* *y*	4 (44.4%)	2 (33.3%)	6 (50.0%)
	≥ *50* *y*	5 (55.6%)	1 (66.7%)	6 (50.0%)
	**Stage at diagnosis**			
	*I – II*	1 (11.1%)	2 (66.7%)	3 (25.0%)
	*III*	2 (22.2%)	0 (0.0%)	2 (16.7%)
	*IV*	5 (55.6%)	1 (33.3%)	6 (50.0%)
	*unknown*	1 (11.1%)	0 (0.0%)	1 (8.3%)
	**ER primary tumor**			
	*Positive*	8 (88.9%)	3 (100.0%)	11 (91.7%)
	*Negative*	1 (11.1%)	0 (0.0%)	1 (8.3%)
	**PR primary tumor**			
	*Positive*	6 (66.7%)	3 (100.0%)	9 (75.0%)
	*Negative*	3 (33.3%)	0 (0.0%)	3 (25.0%)
	**HER2 primary tumor**			
	*Positive*	1 (11.1%)	0 (0.0%)	1 (8.3%)
	*Negative*	8 (88.9%)	3 (100.0%)	11 (91.7%)
	**Grade primary tumor**			
	*I*	0 (0.0%)	0 (0.0%)	0 (0.0%)
	*II*	7 (77.8%)	2 (66.7%)	9 (75.0%)
	*III*	0 (0%)	0 (0.0%)	0 (0%)
	*unknown*	2 (22.2%)	1 (33.3%)	3 (25%)
Metastatic setting	**Distant recurrence free survival**			
	N/A (Stage IV at diagnosis)	5 (55.6%)	1 (33.3%)	6 (50.0%)
	*<10 y*	2 (22.2%)	1 (33.3%)	3 (25.0%)
	≥ *10 y - <20 y*	1 (11.1%)	0 (0.0%)	1 (8.3%-
	>20 y	1 (11.1%)	1 (33.3%)	2 (16.7%)
	**Number of treatment lines**			
	≤ *3*	3 (33.3%)	1 (33.3%)	3 (25.0%)
	> *3 - ≤6*	2 (22.2%)	0 (0.0%)	4 (33.3%)
	> *6 - ≤9*	3 (33.3%)	2 (66.7%)	4 (33.3%)
	> *9*	1 (11.1%)	0 (0.0%)	1 (8.3%)
	*Median number of lines*	6	6	6
Death	**Age at death**			
	< *65* *y*	4 (44.4%)	2 (66.7%)	6 (50.0%)
	≥ *65* *y*	5 (55.6%)	1 (33.3%)	6 (50.0%)
	**Overall survival since diagnosis**			
	< *10* *y*	7 (77.8%)	1 (33.3%)	8 (66.7%)
	≥ *10* *y*	2 (22.2%)	2 (66.7%)	4 (33.3%)

HfO Hope for OTHERS, ER estrogen receptor, PR progesterone receptor, HER2 human epidermal growth factor receptor 2.

In addition to commonly known sites of metastases, such as liver (n = 11/12), bones (n = 7/12) and pleura (n = 5/12), the distinct metastatic pattern of ILC was confirmed with evidence of frequent involvement of the gastro-intestinal tract (n = 7/12), female reproductive organs (n = 5/12) and peritoneum (n = 7/12). Involvement of the lung parenchyma and brain were infrequent (n = 2/12 and n = 3/12), consistent with previous reports^5,20^. Other sites were much rarer such as heart and kidney which were only observed in a single patient. Distribution of the metastases retrieved at autopsy across the different organs per patient is illustrated in Fig. 1B.

### Histopathological characterization of metastatic ILC

**Fig. 2:**
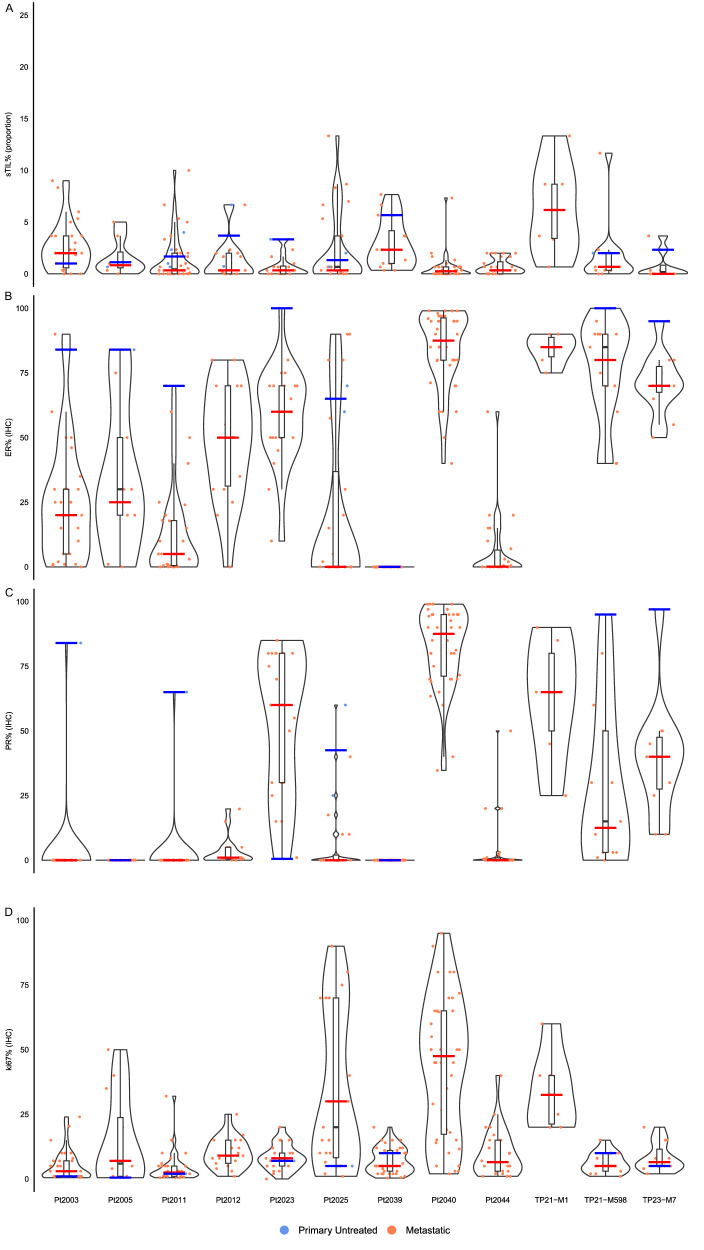
Intra- and interpatient heterogeneity for standard histopathological markers. Intra- and interpatient heterogeneity for sTILs between primary (n = 15 from 9 patients) and metastases (n = 232 from 12 patients) (**A**). Intra- and interpatient heterogeneity for ER between primary (n = 10 from 9 patients) and metastases (n = 265 from 12 patients) (**B**). Intra- and inter-patient heterogeneity for PR between primary (n = 10 from 9 patients) and metastases (n = 264 from 12 patients) (**C**). Intra- and interpatient heterogeneity for KI67 between primary (n = 10 from 9 patients) and metastases (n = 262 from 12 patients) (**D**). Primary samples are illustrated as blue dots and metastases as orange dots. The blue and orange bar indicates the median of the primary and metastatic samples respectively. The primary tumor was not available for all biomarkers for all patients (could not be retrieved or was exhausted). sTIL were scored according to international guidelines, ER and PR according to ASCO/CAP guidelines and KI67 as an average global percentage. sTIL: stromal tumor-infiltrating lymphocytes; ER: estrogen receptor; PR: progesterone receptor.

To explore the immune infiltration in metastatic ILC, sTIL levels were evaluated on 232 metastases and 15 primary untreated tumor samples from core needle biopsies and/or surgical specimens. Ninety (38.79%) metastases had sTIL ≥1%, while 25 (10.78%) had sTIL ≥5%. Across all patients, the median sTIL level in metastases was 0.67% (range: 0-13.33%), while the median for primary untreated tumors was 2% (range: 0.67-6.67%; Fig. 2A). Our regression model showed no evidence for a difference in sTIL levels between primary tumors and metastases (Supplementary Fig. 1). Inter-metastatic comparison of sTIL levels showed significantly higher sTIL levels in axillary lymph node metastases in comparison to all metastases (Estimate: 1.83; 95% CI: 0.25,3.41; p = 0.03; Supplementary Fig. 2, detailed sTIL levels per organ site are presented in Supplementary Fig. 3).

ER-expression was investigated in 265 metastases (examples shown in Supplementary Fig. 4). All but one patient were diagnosed with primary ER-positive ILC. However, 6 patients showed at least one ER-negative metastasis, with a median proportion of ER-negative metastases of 21.88% per patient (range: 5.56-62%) and 5 of them had additionally at least one ER-low (1-10% ER-expression) metastasis. Within one patient, a high variability of ER-expressing cells could be observed (median IQR: 18.75%, Fig. 2B) and the percentage of ER-expressing cells in metastases was significantly lower compared to the primary tumor (Estimate: -49.89; 95%CI: -64.61, -35.16; p < 0.01, Supplementary Fig. 1). Pt2039 was primarily diagnosed with ER-negative disease and at the time of the post-mortem tissue donation all metastases remained ER-negative. Inter-metastatic comparison of ER-expressing cells showed no organ specificity for the percentage of ER-expressing cells (Supplementary Fig. 5).

PR-expression was investigated in 264 metastases (examples shown in Supplementary Fig. 4). Nine patients were primarily diagnosed with PR-positive BC and 6 of them showed at least one PR-negative metastasis with a median of 75.67% PR-negative metastases per patient (range: 8.33-100%, Fig. 2C). Although Pt2023 was primarily diagnosed with PR-negative disease, all but one of the metastases were found to be PR-positive. Intra-patient variability of PR-expressing cells was evident in 7 patients (median IQR: 17.5%) (Fig. 2C) and the percentage of PR-expressing cells in metastases was significantly lower compared to the PR-positive primary tumor (Estimate: –63.08; 95%CI: -87.64, -38.53; p < 0.01, Supplementary Fig. 1). Like ER, there was no organ specific difference in the percentage of PR-expressing cells between metastases (Supplementary Fig. 6).

KI67 was evaluated in 262 metastases (examples shown in Supplementary Fig. 4). The median proliferation index in primary tumors was 5% (range: 0.5-10%) while the median proliferation index in metastases was 8% (range: 0-95%). As shown in Fig. 2D, the proliferation index varied within one patient and the median proliferation index was significantly higher in the metastases compared to the primary tumor (Estimate: 8.23, 95%CI 1.72, 14.74, p = 0.02, Supplementary Fig. 1). Similar to ER and PR, no organ specific increase in the percentage of KI67-expressing tumor cells was observed between metastases (Supplementary Fig. 7).

**Fig. 3:**
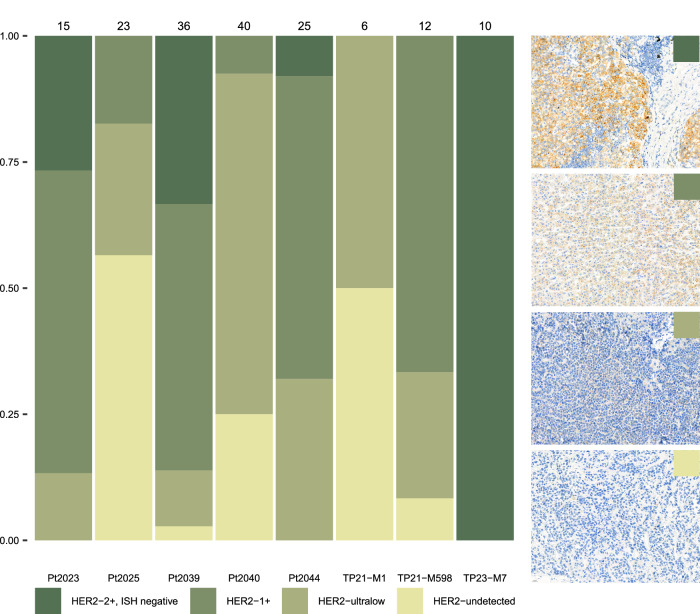
Categorical expression of HER2. HER2 categories from undetected (pale yellow) to HER2 2+ (dark green) in 8 patients with a primary HER2 negative ILC and a total of 167 metastases with adequate FFPE fixation times. All but one patient had metastases that were categorized as HER2-low (HER2 1+ and HER2 2+ in darker green shades), and 7 of the patients had samples that were HER2-ultralow (light green). Five patients presented with HER2-undetectable metastases. HER2 was scored according to ASCO/CAP guidelines. HER2: human epidermal growth factor receptor 2; ISH: In Situ Hybridization.

HER2-expression was assessed in 167 metastases from 8 patients. All but one patient were diagnosed with primary HER2-negative ILC. The metastases sampled at autopsy showed a variety of HER2-staining ranging from HER2-undetected (i.e. HER2-0) to HER2-low (i.e. HER2-1 + , HER2-2 + /ISH negative) (Fig. 3). All but one patient showed HER2-low metastases on immunohistochemistry with a median of 68% metastases per patient (range: 7.5–100%) and 7 patients showed HER2-ultralow (incomplete faint or barely perceptible/weak HER2-staining in <10% of the tumor cells) metastases with a median of 26.09% of metastases per patient (range: 11.11–68%). While the percentage of HER2-expressing cells varied within one patient, there was no significant difference in the percentage of HER2-expressing cells between metastases and primary tumors (Supplementary Fig. 1 and Supplementary Fig. 8). Inter-metastatic comparison showed no organ specific increase in the percentage of HER2-expressing tumor cells (Supplementary Fig. 9).

### Radiological-pathological correlations

**Fig. 4:**
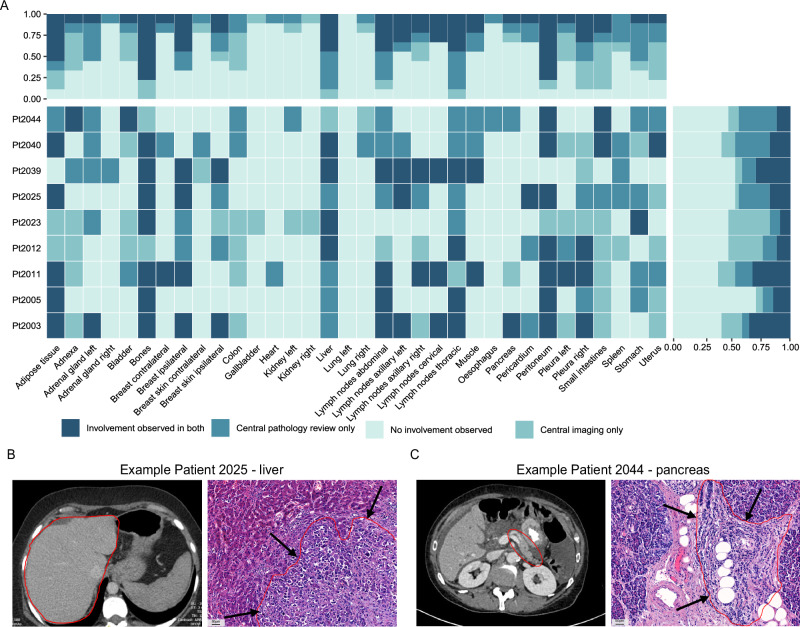
Comparison of autopsy findings with previously performed imaging. **A** Overview of agreement on organ involvement between central imaging review of last available pre-mortem imaging and central pathology review of findings during autopsy. Complete concordance can be identified by the lightest blue (no involvement seen by both) and darkest blue (involvement seen by both) whereas the middle shades indicate absence of concordance to organ involvement. The histogram on top summarizes the proportions of these categories per organ while the histogram on the right summarizes these findings on a patient level. All patients with exception of Pt2011 underwent a CT of thorax and abdomen as last imaging, for Pt2011 a WB-DWI/MRI was performed. **B** Example of a metastasis in the liver of Pt2025 seen by microscopical examination while central imaging review concluded no organ involvement for the liver. Left: liver encircled on CT imaging, Right: HE showing a breast cancer liver metastasis on the right of the red line, highlighted with arrows (20X). **C** Example of a metastasis in the pancreas of Pt2044 seen by microscopical examination while central imaging review concluded no organ involvement. Left: pancreas encircled on CT imaging, Right: highlighted with arrows and in red encircled lobular breast cancer metastasis on HE (20X) CT: computed tomography; WB-DWI/MRI: whole-body, diffusion-weighted MRI; HE: hematoxylin-eosin.

The median time between the last available pre-mortem imaging and post-mortem tissue donation of the 9 UPTIDER patients was 8.7 weeks (range: 1.6-16.7 weeks; Supplementary Fig. 10). The last available scan was a CT scan in eight patients and a WB-DWI/MRI for Pt2011. Involvement of the bones, peritoneum, and adipose tissue was most often annotated in both modalities. Both the central imaging and central pathology review agreed in either organ involvement or no organ involvement in a median of 77.78% organs across all organs (range: 33.3-100% per organ, Fig. 4A). Examples of 100% agreement were the left lung parenchyma as neither the radiologist nor the pathologist detected tumor involvement and right adrenal gland which was not involved in all but one patient.

Central pathology review identified a median of 11.11% organs which were involved but not annotated on imaging (range: 0–44.44% per organ, Fig. 4A). The involvement of left adrenal gland, spleen, thoracic lymph nodes and liver were more often observed upon microscopic pathological examination and not on imaging suggesting lower resolution of CT for these sites. However, this was also observed for the bladder, heart, liver, stomach and uterus in Pt2011 for whom a WB-DWI/MRI was performed. Two examples of organ involvement identified only by central pathology are presented in Fig. 4B, C. On the other hand, central imaging review also detected a median of 11.1% organ involvement which was not confirmed at the pathology level (range: 0–55.56% per organ, Fig. 4A). The involvement of adnexa, uterus, small intestines and colon was more often seen on imaging but could not be confirmed on pathological review because a biopsy either did not show any tumor upon microscopic evaluation or no biopsy was taken during autopsy because the site was not suspicious on gross inspection.

At the patient level, the radiologist and pathologist agreed on a median of 70.59% organs either being involved or not (range: 55.88–85.29% per patient, Fig. 4A). For example, there was a high agreement for Pt2005. Central pathology review identified independently a median of 11.76% organs involved (range: 5.88–32.35% per patient, Fig. 4A). For example, the pathologist detected more organ involvement in Pt2044 such as abdominal lymph nodes, stomach and uterus. However, central imaging review revealed independently a median of 11.8% involved organs (range: 2.94–35.29% per patient, Fig. 4A). For example, there was more involvement detected on central imaging review in Pt2023 as the involvement of for example adipose tissue, adnexa, bladder and colon was only detected by the radiologist.

## Discussion

The post-mortem tissue donation programs presented here, are, to our knowledge, the only programs that have specific research questions dedicated to metastatic ILC. This resulted in an unprecedented and methodical sampling of ILC metastases, providing a great opportunity to study the intra- and inter-patient heterogeneity of standard BC biomarkers and to compare the metastatic pattern via imaging and histopathology.

Most patients were diagnosed with a grade 2, strongly ER/PR positive and *HER2* non-amplified primary ILC consistent with documented clinical characteristics^10^. Half of the patients in our cohort were diagnosed with classic ILC, before the age of 50 and had de novo metastatic disease at initial diagnosis (Supplementary Table 1). This small cohort might not be representative of the general population of patients with ILC which tends to be older at diagnosis and from whom only 13% shows stage IV disease at diagnosis^2,21^. ILC has been associated with late to very late recurrences^2^. This was reflected by 2 patients in our cohort presenting with recurrences after more than 20 years.

Metastatic sites that are more associated with ILC than other subtypes of BC include the peritoneum, stomach, intestines, reproductive organs and adrenal glands^3,5,6,20^. All metastatic sites were frequently involved in our cohort confirming the distinct metastatic spread of ILC. Other rare sites that were also involved included for example lung, muscle and bladder^5,22,23^. Although ILC tends to show more frequently leptomeningeal metastases compared to brain metastases, only brain was involved in a minority of our cohort^5^.

Regarding the biomarkers, we first evaluated sTIL. Reduced sTIL levels have been consistently reported in metastases compared to primary tumors in several observational studies that evaluated BC in general^24–26^. In our series, we observed that the median sTIL levels were very low ( ≤5%) both in the primary tumor as well as in the metastases, with no significant difference between the two. This is in line with the results of Richard et al. where they also observed low sTIL levels without statistical evidence in metastatic biopsies taken as compared to the matched primary tumor in 67 patients with ILC^24^. Recently, combination immunochemotherapy was tested in metastatic ILC within the GELATO trial. However, clinical benefit was demonstrated in only 6 of the 23 patients included (26%, with 4 partial responses and 2 stable diseases, 4 of them with triple negative breast cancer (TNBC))^27^. The authors also could not detect an association between immune infiltration and clinical benefit^28–30^. In our series of patients with ILC, we only had one patient with TNBC ILC (Pt2039) who was de novo metastatic and treated with pembrolizumab which was terminated after 6 cycles due to disease progression. While Richard et al. did not observe differences in sTIL levels according to the organ, we observed higher sTIL levels in axillary lymph nodes as compared to the other metastatic sites. We want to reiterate that we only scored sTIL in lymph nodes with complete replacement of the lymphoidal tissue or in the regions with extranodal extension. Previous reports have also suggested that in patients with metastatic BC, higher levels of immune infiltration can be found in axillary lymph node biopsies as compared to other metastatic regions^31,32^. Rye et al. analyzed the immune cells present in sentinel lymph node metastases and observed an high levels of regulatory T cells as well as CD8 T cells, and especially exhausted CD8 T cells. These observations could imply that these axillary lymph node metastases serve to elicit immune suppression^33^. In addition to the axillary lymph nodes, our inter-organ comparison also revealed a non-significant trend toward increased sTIL levels in thoracic lymph nodes. This raises the possibility that the tumor may exploit these sites to promote broader systemic immune suppression as a survival strategy. To date, there is no published literature specifically addressing the immune response in distant lymph nodes. The relevance of sTIL levels and their exact composition and phenotype in each organ site needs to be further studied to understand the potential implications in the treatment of metastatic ILC.

Regarding the hormone receptors, while loss of expression of ER and PR in metastases originating from ER/PR-positive primary tumors has been extensively described for BC in general^28,29,34–36^, the evidence for ILC is much scarcer^9^. In our cohort of heavily pretreated patients, more than half of the patients with a primary ER- or PR-positive tumor had at least one ER- or PR-negative metastasis, respectively. Additionally, we showed that the percentage of ER- and PR-expressing cells was significantly lower in metastases compared to the primary tumor. Overall, our observations, which are based on multiple metastatic samples per patient, as opposed to previously reported single-metastasis based studies, suggest that the loss of or a decreased ER and PR-expression as mechanisms of treatment resistance is more frequent than previously reported. To enhance our understanding of the different endocrine resistance mechanisms and linked inter-metastatic heterogeneity that exist in these patients with primary HR + ILC treated with endocrine therapy, we will be conducting DNA sequencing analyses to assess for the presence of genomic alterations associated with endocrine resistance^30^.

Regarding HER2, we observed that all patients had at least one metastasis that could be categorized as HER2-low or HER2-ultralow. Therefore, many of these patients would be eligible to receive trastuzumab-deruxtecan^37^. However, as previously mentioned in our report on intra-patient heterogeneity of HER2 status, one biopsy might not be emblematic for the entire disease and might thus not be suitable to influence patient selection for this treatment^38^. Here, 3 patients had all metastases being HER2-low or HER2-ultralow. For these patients, it would therefore not matter which metastasis would be biopsied. For the other 5 patients, the percentage of HER2-low or HER2-ultralow metastases ranged between 40% and 97%, suggesting that a second biopsy might be recommended in case the first one would report a HER2-undetected (i.e. HER-0) metastasis.

Regarding KI67, in agreement with some studies, a significantly higher level of KI67 was observed in the metastatic samples compared to the primary samples^39^. While the prognostic value has mainly been studied in primary BC, several studies have shown that high expression levels of KI67 in metastatic biopsies are associated with poorer survival outcomes^39–41^, although the association with survival did not remain significant in multivariable analyses^41^. The higher levels of KI67 expression observed in ILC metastases as compared to the primary tumor may suggest that it might be worth to rechallenge the disease with therapies targeting cell proliferation, although the clinical relevance still needs to be assessed.

To conclude the part on the biomarkers, based on our current findings on metastatic ILC and our previously published report on intra-patient heterogeneity in metastatic BC, it is evident that a single biopsy insufficiently captures the complexity of metastatic disease^38,42^. The 6^th^ and 7^th^ International Consensus Guidelines for the management of advanced BC recommend a biopsy of newly diagnosed lesions, where feasible, to confirm metastatic disease. However, these guidelines only suggest biomarker reassessment “at least once“^43^. The intra-patient heterogeneity of our cohort warrants reassessment of ER, PR and HER2 every time another metastasis is biopsied to provide the patient with the opportunity of receiving the most appropriate treatment(s). In the literature, treatment modifications are however reported to be implemented in only up to 20% of BC cases displaying changes in biomarker expression^44,45^. In the future, biomarker assessment through liquid biopsies or biomarker-specific imaging could offer a superior alternative to single metastatic biopsies. However, these approaches remain under investigation and require thorough validation before they can be implemented in clinical practice.

Regarding our second objective, close collaboration with the radiologist provided us with the unique opportunity to compare the result of our extensive sampling at autopsy with radiology findings from the last available pre-mortem imaging. The relatively high median agreement on either organ involvement or no organ involvement of nearly 78% across all evaluated organs was contrasted by some organs with low agreement. The most striking example was provided by the liver which by imaging and gross inspection, showed no signs of invasion while upon microscopic examination liver metastases were present in 4 out of the 8 patients (50%). This could be explained by the lack of mass formation on imaging and random sampling of the organ during post-mortem tissue donation procedure. On the other hand, involvement of the uterus was more often reported by the radiologist but was missed on microscopic examination, which might suggest sampling bias due to normal appearance of the organ on gross examination. The radio-pathological discordance in these cases can be also explained by the lack of additional random biopsies because of the uncommon involvement of the uterus as compared to liver in metastatic BC possibly justifying a less extensive sampling approach.

Although it is known that the monitoring yield of a CT scan can be compromised by the specific growth pattern of ILC^4,46^, it was still the most used imaging modality in our cohort. Additionally, the anatomical resolution of CT scans might also not be high enough to distinguish between peritoneal/omental lesions on specific organs like intestines, uterus and bladder and clear infiltration of these organs^47^. This might explain why certain organ sites were seen as involved on imaging, while gross inspection and the microscopical findings afterwards resulted in the involvement being attributed to another tissue/organ. While WB-DWI/MRI is believed to overcome several of the limitations of CT^47^, it was only performed in one patient so no conclusions can be drawn on its ability to detect metastatic ILC lesions specifically. However, in terms of pathological examination, gross inspection revealed no clear organ involvement in some cases while random sampling revealed microscopic infiltration by ILC, as previously described^19^. However, not all organs were randomly sampled and random sampling by itself might miss infiltration of the organ at a different location. As illustrated above, the involvement of the uterus was more often missed by central pathology review and an expansion of our random sampling procedure might reveal additional invasion of for example the uterus. The comparison between radiology and pathology was further limited by the interval between last imaging and death (median interval: 8.7 weeks) since we cannot exclude further progression of the disease during this window of time. Therapeutic response might look suspicious on imaging and might have been recorded as organ involvement, however without the presence of tumor remnants microscopical evaluation concluded no involvement. We hypothesize that the disease extent of ILC is underestimated by CT scan and thus, improved imaging modalities are urgently needed for patients with metastatic ILC. Research on imaging improvements was also ranked as a key priority by patients with ILC in a recent survey^48^. Additionally, more insights are needed to determine the complementary value of liquid biopsies to detect disease progression and to reflect extent of disease in patients with ILC specifically.

This study has several limitations, some of which are inherent to the post-mortem setting. Although only 12 patients were included, comprehensive sampling of metastases resulted in an unprecedented number of metastatic ILC samples. All patients had undergone extensive treatment, with varying regimens, which may have influenced marker expression levels in the metastases. The use of post-mortem tissue is associated with prolonged cold ischemia times; however, tissue donation was performed as promptly as possible. Previous findings from our group have demonstrated that protein expression remains stable within the first 11 hours after death^19,42^. In comparing imaging and pathological findings, it is important to note that tissue samples were collected only from metastases, suspicious organs, and randomly selected organs commonly infiltrated by ILC. Consequently, microscopic infiltration in some organs may have been missed. In contrast, radiological assessments classified any suspicious lesion as involved, which may have led to false-positive interpretations.

To conclude, we were able to collect an unprecedented number of metastatic samples from patients with ILC and confirmed intra- and inter-patient heterogeneity of standard histopathologic biomarkers. This has important clinical implications: a single biopsy with the purpose of detecting targetable alterations in patients with metastatic cancer, in the context of ILC, might not reflect the entire metastatic disease. Since HER2-low and/or HER2-ultralow metastases were found in all patients, we believe that trastuzumab-deruxtecan should be considered in these patients. Furthermore, we have confirmed that ER and PR levels are significantly reduced while KI67 levels are increased in ILC metastases compared to primary samples. Lastly, although in a small cohort, fair agreement concerning organ involvement per organ between imaging by CT scan and pathologic examination was frequent, suggesting that efforts are needed to improve imaging modalities for patients with metastatic ILC.

## Methods

### Patient enrollment and sample collection

This study is a collaboration between two monocentric post-mortem tissue donation programs, UPTIDER, Belgium (clinicaltrials.gov: NCT04531696, registered 11th of august 2020) and HfO, Pittsburgh, USA. Both programs are in accordance with the Helsinki Declaration of 1964, as amended most recently in 2008, and enroll consenting patients with metastatic BC. Both studies were approved by the respective ethical committees of each center (UZ Leuven: S64410, University of Pittsburgh: STUDY19060376). Written informed consent was obtained from each of the participants. Primary tumor samples are retrieved after patient inclusion. Both in UPTIDER and HfO, tissues with clear gross infiltration were sampled and fixed in formalin during autopsy. Additionally, macroscopically normal-looking organs were randomly sampled in UPTIDER. Information regarding patient inclusion in UPTIDER and HfO, sample collection and data curation of patient information are present in the original publications^19,49^. For this study, all patients diagnosed with primary ILC, regardless of the ILC subtype, who have undergone post-mortem tissue donation before June 30, 2024 are reported here. Information on treatment is extracted from patient files. In the metastatic setting, a new treatment line is assigned when disease progression is documented^50^.

### Histopathological characterization

Histopathological characterization was performed on all formalin-fixed paraffin embedded (FFPE) samples obtained during the autopsy and on the untreated primary tumor samples. A primary tumor was considered untreated if the patient did not receive any systemic/localized cancer treatment in the chest-area within 5 years before the breast cancer diagnosis. Pt2012, had a prior history of a pT1N1b IBC-NST before being diagnosed with contralateral ILC 14 years later. Here, the contralateral tumor with ILC histology was regarded as the primary, untreated tumor. The histological subtype of the primary tumor samples was centrally reviewed by a board-certified pathologist with over 10 years’ experience in reporting BC (G.F). The primary tumor sample of Pt2044 was not retrievable, therefore we relied on the historic pathology report for the histological subtype of the primary tumor. All samples were centrally scored on hematoxylin-eosin by G.Z. and G.F. in UZ Leuven for tumor cellularity. sTIL were assessed according to the (adapted) international guidelines in three representative fields and the average was computed^51–53^. For lymph nodes, sTIL scoring was only performed if the tissue was entirely replaced by tumor or in regions with extra-nodal extension. In bone metastases, sTIL scoring was only performed when the bone marrow was completely replaced by tumor or in case the metastasis involved surrounding soft tissue beyond the bone cortex. Spleen metastases were excluded for sTIL analyses. In liver metastases, sTIL scoring was adapted from the international guidelines and were scored along the tumor-liver interface while considering for tumor architecture and growth patterns^54^. Bone samples are decalcified using unbuffered 10% ethylenediaminetetraacetic acid (EDTA). On all samples with >10% tumor cellularity, immunohistochemistry (IHC) was performed for ER, PR, KI67 and HER2. The antibodies used for immunohistochemistry were: ER (antibody clone EP1, DAKO, RTU, CE-IVD, Santa Clara, United States), PR (1294, DAKO, RTU, CE-IVD, Santa Clara, United States), KI67 (MIB1, DAKO, RTU, CE-IVD, Singapore) and HER2 (anti-human c-erbB-2 protein, DAKO, 1:200, Singapore). Two patients from the UPTIDER-program were excluded from the HER2 analysis because of inadequate FFPE fixation times of >20 days (Pt2011, Pt2012) and HER2-scoring was not available for one additional patient at the time of writing the manuscript (Pt2005). As described by Arber et al., staining performance of ER, PR and KI67 staining are less sensitive with longer fixation times^55^ allowing us to keep them for analysis of ER, PR and KI67. ER and PR were scored according to the ASCO/CAP guidelines of 2020 with cut-off for positivity of 1%^56^. KI67 score was determined by estimating the average percentage of positively stained tumor cell nuclei among the total number of tumor cell nuclei across the entire sample. HER2 was assessed according to ASCO/CAP guidelines, as previously described^38,57^. HER2-0 samples per ASCO/CAP guidelines were categorized either as HER2-undetected as these samples do not show protein expression, or as HER2-ultralow category when incomplete faint or barely perceptible/weak HER2-staining in <10% of the tumor cells was observed. HER2-low was assigned to samples displaying faint/barely perceptible partial, membranous staining in >10% tumor cells (HER2 1 + ) and weak to moderate complete membranous staining in >10% of tumor cells and negative on fluorescent in-situ hybridization (FISH; HER2-2+ , FISH negative). Of note, in some cases, tumor samples could not be scored in case of exhaustion of the block, lack of remaining tumor on the section or technical artefact.

### Radiological-pathological correlations

The last available pre-mortem CT or WB-DWI/MRI before death from nine UPTIDER patients was reviewed by the radiologist (R.D.). The findings of the autopsy were blinded for the radiologist. A predefined list of organs was provided that was scored to be affected by metastases (‘organ involvement’) or not (‘no organ involvement’) both by the radiologist and with the results of the microscopical evaluation from all samples taken during autopsy. If an organ looked suspicious/showed suspicious areas on imaging although metastatic involvement was unclear, it was considered as ‘organ involvement’. If no sample was taken from an organ during autopsy, this was considered as ‘no organ involvement’ for central pathology review. Results per organ from both imaging and pathology were then compared and categorized as “involvement observed in both”, “central pathology review only”, “central imaging only” and “no involvement observed”.

### Statistical Analysis

Due to the unique nature of sample collection, a detailed outline of the biological information offered by each unique tumor was essential. Hence, we implemented a methodology that used metadata at the sample level such as organ localization, site of organ collection and sample type to consolidate the multiple samples collected per lesion into designated unique lesions: metastases and primary untreated tumors. ER%, PR%, KI67%, and HER2% corresponding to these unique tumors were calculated using weighted average considering the tumor cellularity per sample along with their independent expression levels (%). To consolidate the sTIL levels for unique tumors, a mean value of all samples per unique lesion was ascertained as the representative sTIL level for each unique tumor. Regression analysis was performed to fit histopathological parameters between groups of interest (metastases vs primary untreated tumors and metastases of interest vs all metastases). Associations between the outcome variable of interest (sTIL levels, ER%, PR%, KI67%, HER2%) and independent co-variates (groups of interest) were assessed by linear mixed quantile regression with a random effect on patient ID. The analysis was done using the lqmm package (v.1.5.8) on R (v.4.3). All plots were designed using the ggplot2 package (v.3.4.3).

To study the degree of organ involvement between central imaging and central pathology, binary scores of 0 and 1 were allotted to lesions observed on central imaging and microscopically during central pathology review, with scores from central pathology taken as reference. As such, 4 categories were ascertained based on the possible combinations: No involvement observed (0_0), Central imaging only (0_1), Central pathology review only (1_0) and Involvement observed in both (1_1). No formal testing was performed for statistical evidence to complement the results of this analysis.

## Supplementary information

Supplementary information

## Data Availability

The histopathological data underlying this article will be available in a CodeOcean repository with the following 10.24433/CO.7065184.v1.
