# Supplementary material for: Clinical and histopathological characterization of metastatic lobular breast cancer: lessons learned from post-mortem tissue donation programs
Source: NPJ Breast Cancer. 2026 Feb 20;12:48. doi: 10.1038/s41523-026-00912-5 (PMC13036049; doi:10.1038/s41523-026-00912-5)
Supplement: Supplementary file 1 — Supplementary information [file 41523_2026_912_MOESM1_ESM.pdf]

Supplementary: Clinical and histopathological characterization of metastatic lobular breast cancer:

lessons learned from post-mortem tissue donation programs

G. Zels, K. Van Baelen, A. C. C. Chang, et al.

Supplementary tables

Supplementary Table 1

PatientID	Surrogate molecular subtype	Age at diagnosis (years)	DRFS (months)	OS (months)	Breast surgery	Neoadjuvant therapy	Adjuvant therapy	Metastatic treatment line 1	Metastatic treatment line 2	Metastatic treatment line 3	Metastatic treatment line 4	Metastatic treatment line 5	Metastatic treatment line 6	Metastatic treatment line 7	Metastatic treatment line 8	Metastatic treatment line 9	Metastatic treatment line 10
2003	ER+/PR+/HER2+	80	0	47	no	no	NA	paclitaxel + trastuzumab + pertuzumab, letrozole	trastuzumab-emtansine	radiotherapy, fulvestrant	letrozole + lapatinib						
2005	ER+/PR-/HER2-	53	55	79	mastectomy + axillary clearance	no	epirubicin + cyclophosphamide, paclitaxel, radiotherapy, letrozole	fulvestrant + palbociclib	trial drug: tesetaxel	intrathecal methotrexate, cytarabine, hydrocortison							
2011	ER+/PR+/HER2-	51	75	106	mastectomy + axillary clearance	no	5-fluorouracil, epirubicin, cyclophosphamide + taxotere, radiotherapy, letrozole with switch to tamoxifen	bilateral salpingo-oopherectomy, trial: fulvestrant + palbociclib	exemestane + everolimus	capecitabine	vinorelbine, radiotherapy	liposomal doxorubicin + cyclophosphamide, radiotherapy	carboplatinum + gemcitabin, radiotherapy				
2012	ER+/PR+/HER2-	37	358	427	tumorectomy + axillary clearance	no	radiotherapy, tamoxifen	tamoxifen	letrozole	fulvestrant	trial: paclitaxel + IMP321, liposomal doxorubicin + cyclophosphamide, capecitabine	letrozole + abemaciclib	liposomal doxorubicin + cyclophosphamide	carboplatin + gemcitabin	vinorelbine	exemestane + everolimus	

2023	ER+/PR-/HER2-	70	0	83	no	NA	NA	trial: fulvestrant + ribociclib	tamoxifen, trial: elacestrant	radiotherapy, letrozole	paclitaxel, exemestane	capicitabine, vinorelbine	eribulin	carboplatin + gemcitabin	epirubicine		
2025	ER+/PR+/HER2-	44	0	15	no	NA	NA	bilateral salpingo-oopherectomy, trial: placebo + letrozole + palbociclib	capecitabine	carboplatinum + 5-fluorouracil, paclitaxel							
2039	ER-/PR-/HER2-	83	0	19	no	NA	NA	pembrolizumab	capecitabine	cyclophosphamide, radiotherapy							
2040	ER+/PR+/HER2-	49	0	55	mastectomy + axillary clearance	NA	NA	letrozole + palbociclib, bilateral salpingo-oopherectomy, radiotherapy	trial: camizestrant	exemestane + everolimus	capecitabine	paclitaxel	eribulin	epirubicin + cyclophosphamide			
2044	ER+/PR+/HER2-	47	192	325	tumorectomy + axillary clearance	NA	radiotherapy, tamoxifen	tamoxifen, mastectomy, radiotherapy	letrozole	fulvestrant + palbociclib, radiotherapy	exemestane + everolimus	capecitabin	paclitaxel	trial: eribulin	liposomal doxorubicin + cyclophosphamide	carboplatinum + gemcitabin	vinorelbine
TP21-M1	ER+/PR+/HER2-	50	261	301	mastectomy	NA	tamoxifen	anastrozole	fulvestrant	palbociclib + letrozole	exemestane + everolimus	tamoxifen	capecitabin	paclitaxel	doxorubicin		
TP21-M598	ER+/PR+/HER2-	38	0	28	no	NA		goserelin, letrozole, paclitaxel, palbociclib	fulvestrant + alpelisib, capecitabine, tamoxifen								
TP23-M7	ER+/PR+/HER2-	48	116	190	mastectomy	NA	tamoxifen	letrozole + palbociclib	anastrozole + palbociclib	fulvestrant + palbociclib	fulvestrant + abemaciclib	capecitabin	trial: radium-223 dichloride with paclitaxel	trastuzumab-deruxtecan, tamoxifen	exemestane, alpelisib		

ER: estrogen receptor; HER2: human epidermal growth factor receptor 2; NA: not applicable; PR: progesterone receptor

Supplementary figures

Supplementary Figure 1

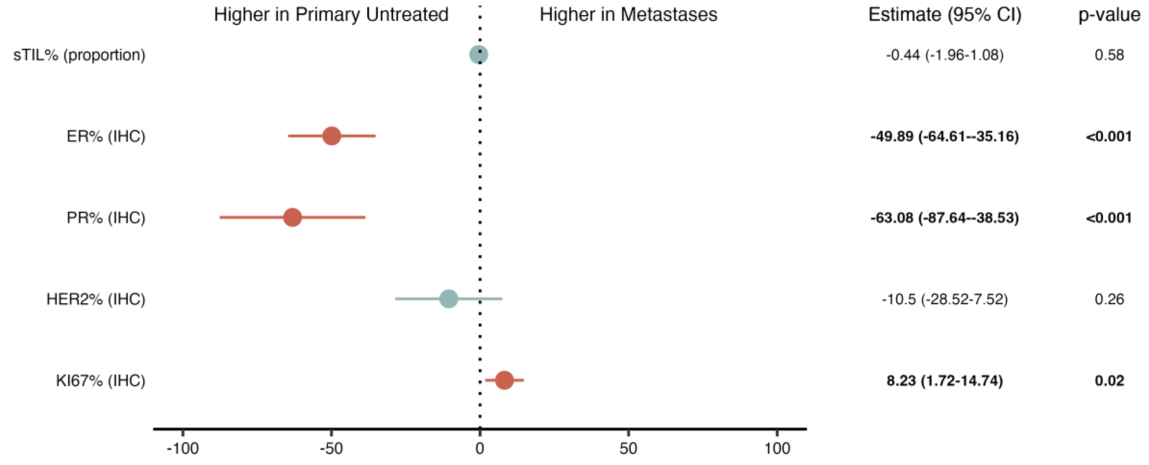

Forest plot depicting the median change in biomarker levels between metastases and primary untreated tumors across the patient cohort. Segments in red indicate statistical significance (p<0.05). p-values were derived using two-sided Wald's test. sTIL = stromal tumor-infiltrating lymphocytes, ER = estrogen receptor, PR = progesterone receptor, HER2 = human epidermal growth factor receptor 2

Supplementary Figure 2

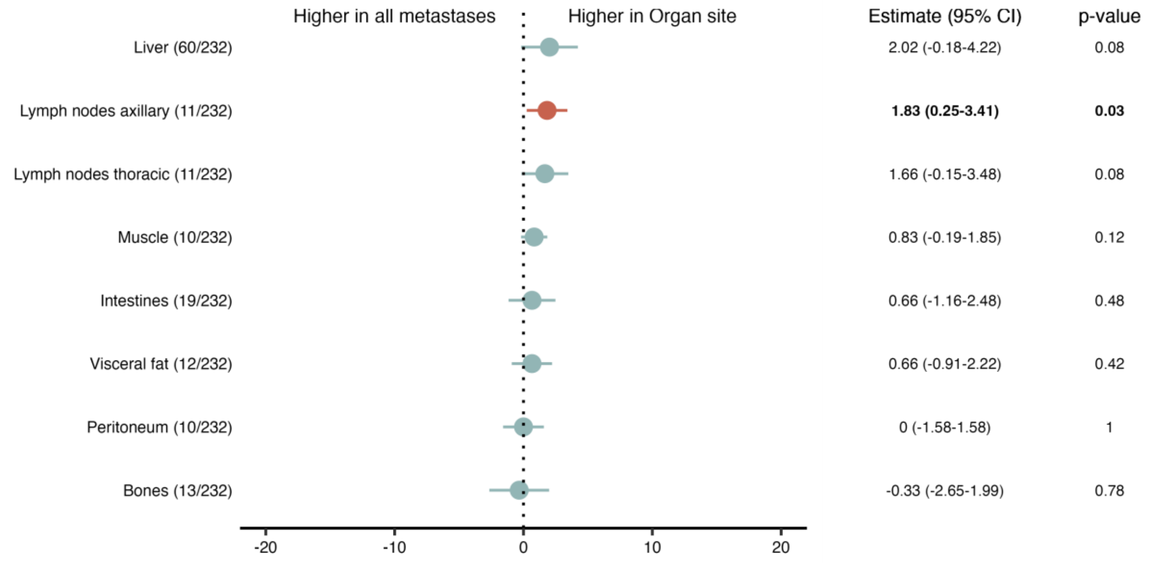

Forest plot depicting the median change in sTIL levels between organ sites that had at least 10 lesions and all metastases across the patient cohort ranked from lowest to highest change in point estimate. Segments in red indicate statistical significance (p<0.05). p-values were derived using two-sided Wald's test. sTIL = stromal tumor-infiltrating lymphocytes

Supplementary Figure 3

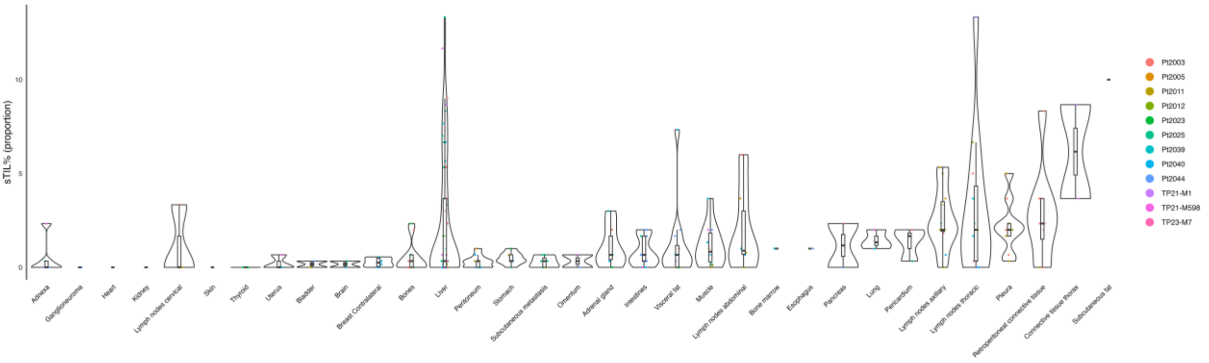

Violin plot illustrating the sTIL levels per organ

Supplementary Figure 4

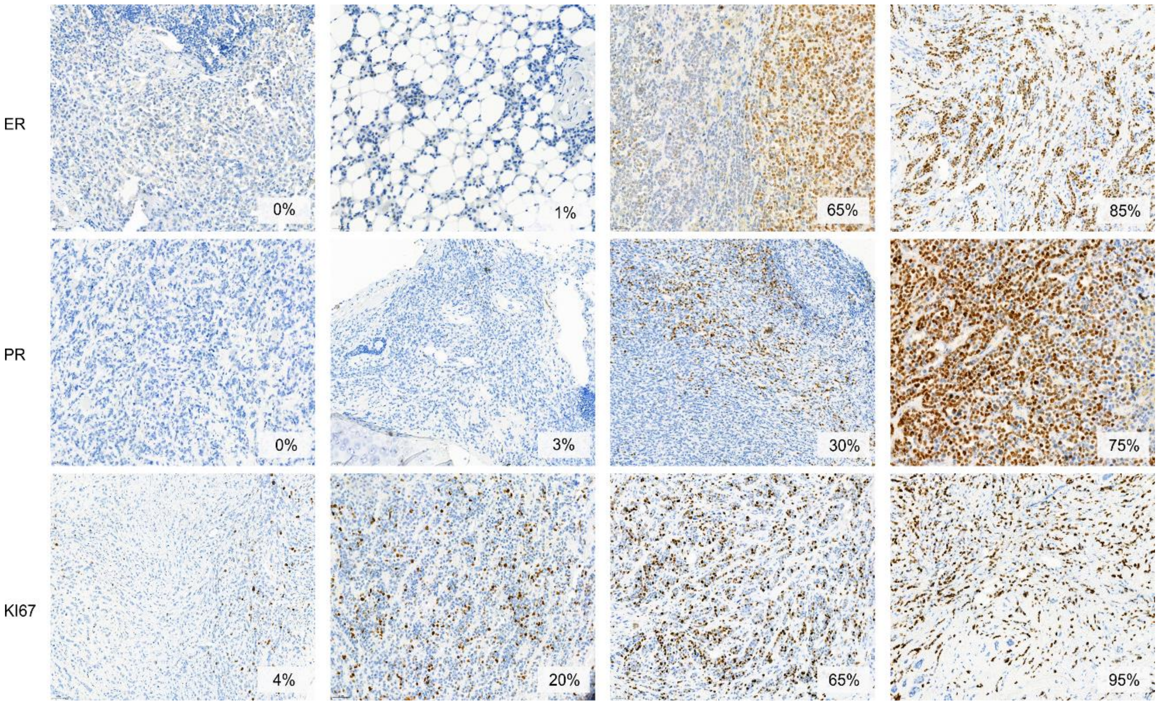

Examples of ER, PR and KI67 staining. ER and PR were scored according to ASCO/CAP guidelines with the cut-off for positivity set at 1%. KI67 was scored as a global average percentage. All pictures were taken at 25X magnification. ER: estrogen receptor; PR: progesterone receptor; ASCO/CAP: American Society of Clinical Oncology/College of American Pathologists

Supplementary Figure 5

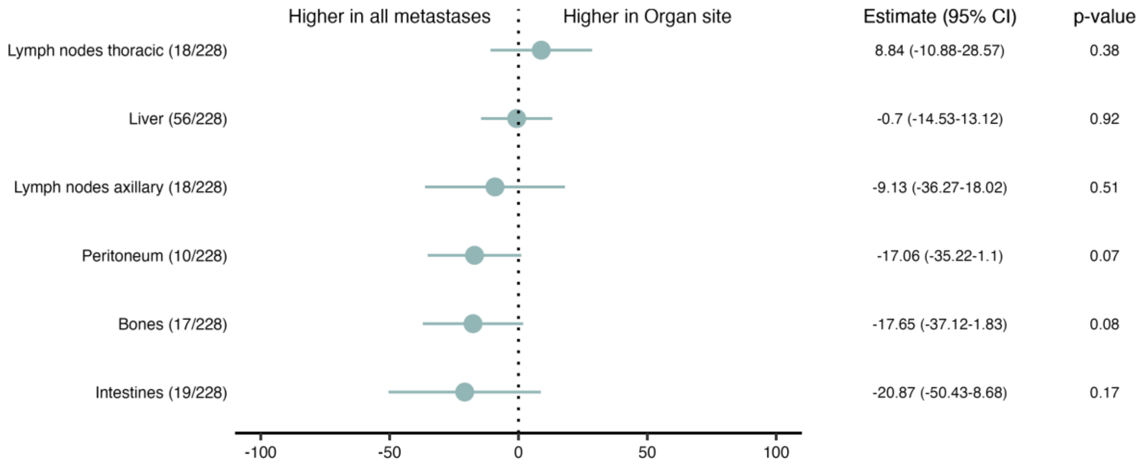

Forest plot depicting the median change in ER levels between organ sites that had at least 10 lesions and all metastases in patients with ER+ primary disease, ranked from lowest to highest change in point estimate. Segments in red indicate statistical significance ($p<0.05$). p-values were derived using two-sided Wald's test. ER = estrogen receptor

Supplementary Figure 6

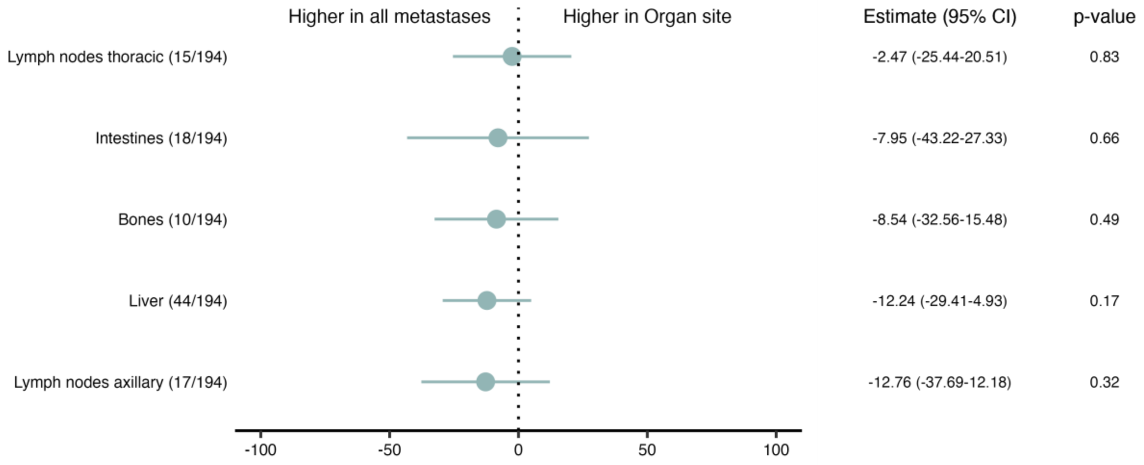

Forest plot depicting the median change in PR levels between organ sites that had at least 10 lesions and all metastases in patients with PR+ primary disease ranked from lowest to highest change in point estimate. Segments in red indicate statistical significance ($p<0.05$). p-values were derived using two-sided Wald's test. PR = progesterone receptor

Supplementary Figure 7

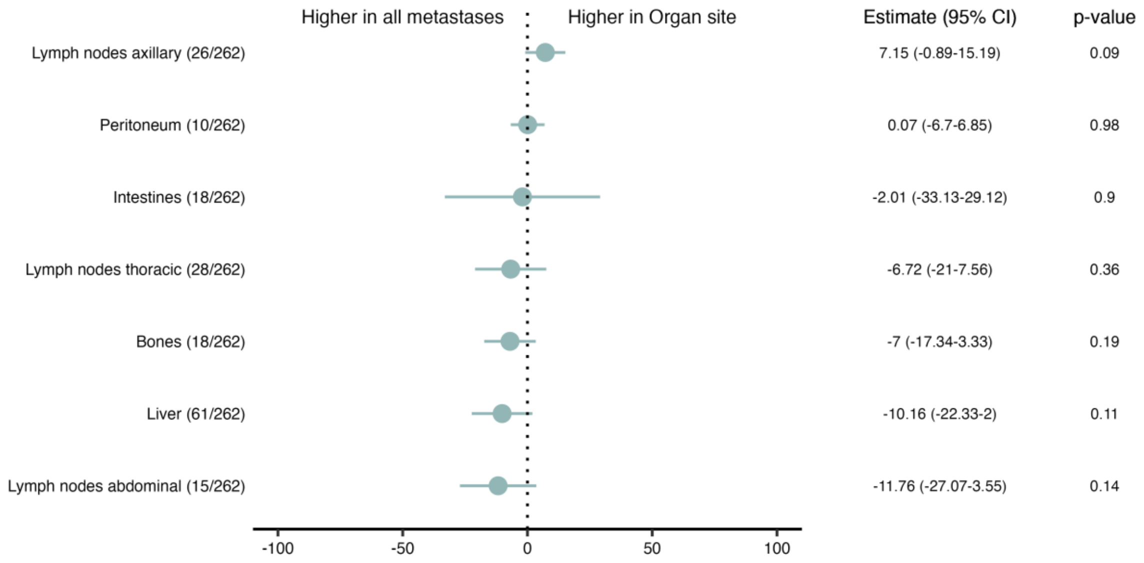

Forest plot depicting the median change in KI67 levels between organ sites that had at least 10 lesions and all metastases across the patient cohort ranked from lowest to highest change in point estimate. Segments in red indicate statistical significance (p<0.05). p-values were derived using two-sided Wald's test.

Supplementary Figure 8

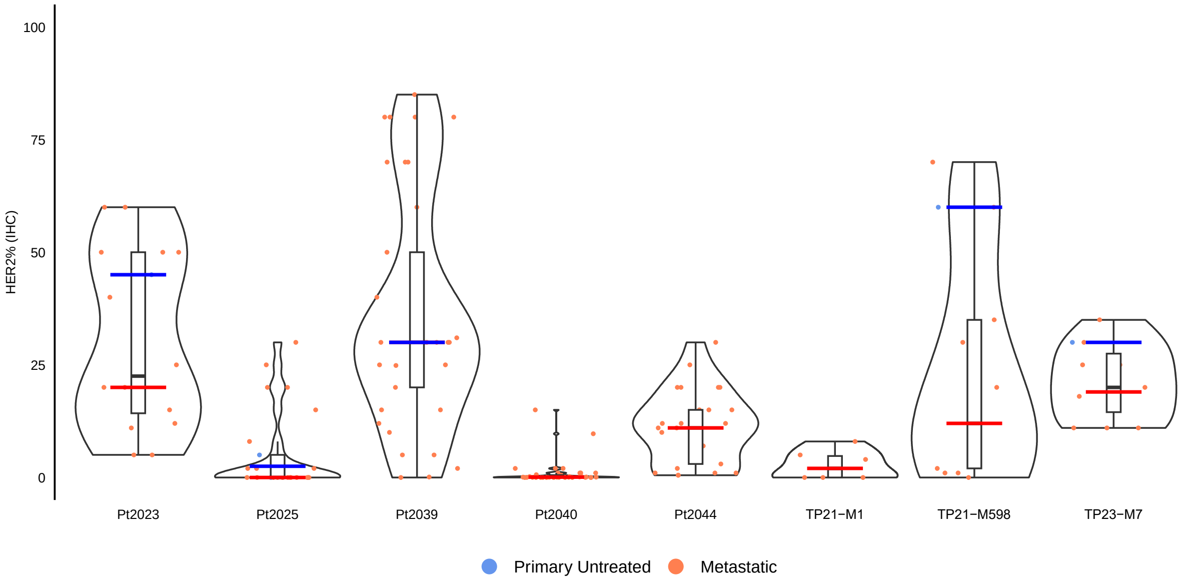

Violin plot depicting the intra- and inter-patient heterogeneity for HER2 expressing cells between primary and metastatic samples. Primary samples are illustrated in blue dots and metastases in orange dots; the blue and orange bar indicates the median of the primary and metastatic samples respectively. Only the samples from eight patients had a fixation time of <28 days and were included for HER2 immunohistochemistry. HER2 = human epidermal growth factor receptor 2

Supplementary Figure 9

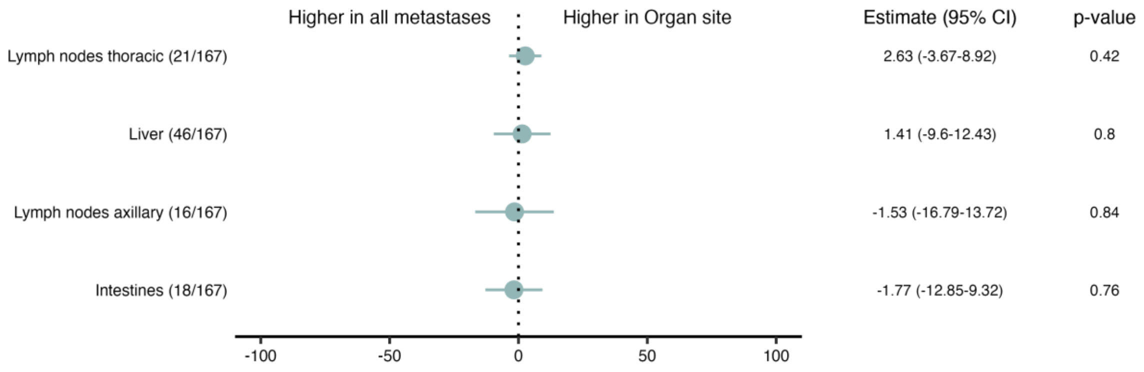

Forest plot depicting the median change in HER2 levels between organ sites that had at least 10 lesions and all metastases in patients with HER2- disease ranked from lowest to highest change in point estimate. Segments in red indicate statistical significance ($p<0.05$ p-values were derived using two-sided Wald's test. HER2 = human epidermal growth factor receptor 2

Supplementary figure 10

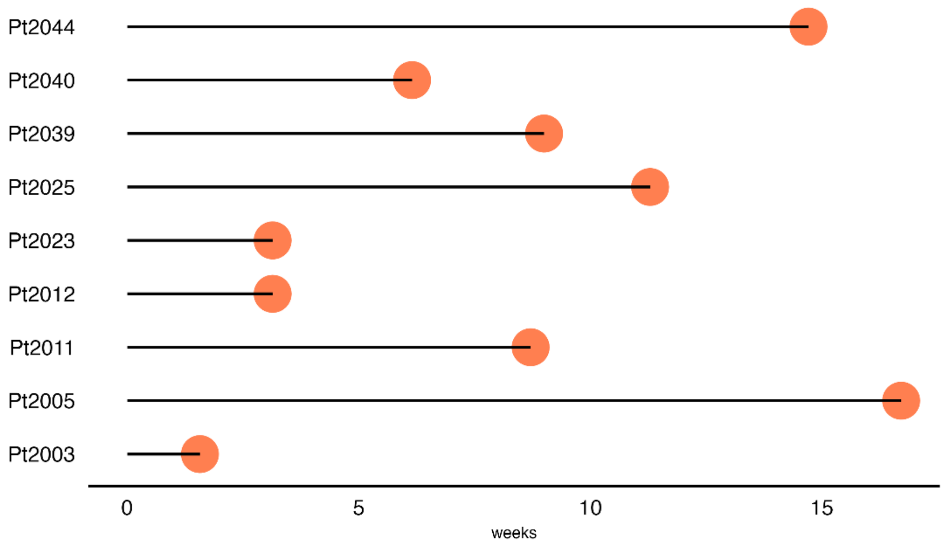

Time in weeks from last available imaging to autopsy.
